# Antisynthetase Syndrome Post Shingrix and Pneumovax Vaccinations, Possible Correlation

**DOI:** 10.7759/cureus.25085

**Published:** 2022-05-17

**Authors:** Alsayed Osman, Ahmad Almusa, Robert Ryad, Bahar Sumbulyuksel

**Affiliations:** 1 Internal Medicine, AdventHealth Orlando, Orlando, USA; 2 Rheumatology, AdventHealth Orlando, Orlando, USA

**Keywords:** interstitial lung disease (ild), anti-jo-1 antibody, autoimmune disease, vaccination, antisynthetase syndrome (ass)

## Abstract

This is a case report of a patient who developed acute progressive shortness of breath that started two days following the administration of Shingrix and Pneumovax vaccinations. Eight days after the onset of his symptoms he was diagnosed with acute interstitial pneumonitis based on CT scan of the chest which later appeared to be consistent with the diagnosis of antisynthetase syndrome in light of findings consistent with mechanic's hands on examination, elevated Anti-Jo-1 antibody titers and aldolase on laboratory studies.

## Introduction

Antisynthetase syndrome (ASS) is an autoimmune disease characterized by autoantibodies against one of many aminoacyl transfer RNA (tRNA) synthetases with clinical features that may include interstitial lung disease (ILD), non-erosive arthritis, myositis, Raynaud's phenomenon, unexplained fever, and mechanic's hands [1]. Anti-Jo-1 antibody was the first to be discovered and most commonly identified among the autoantibodies, and is present in approximately 30% of cases [2].

It was not until 2010 that formal criteria for the diagnosis of ASS were introduced by Connors et al. [1]. These criteria proposed that all patients with ASS must have evidence for a tRNA synthetase autoantibody, in addition to one or more of the following clinical features: mechanic’s hands, Raynaud’s phenomenon, myositis, interstitial lung disease (ILD), arthritis, and unexplained fever. In 2011, Solomon et al. proposed alternative, stricter criteria, requiring two major or one major and two minor criteria, in addition to the presence of an aminoacyl tRNA synthetase autoantibody [3].

ILD is the most common extra-muscular manifestation with a prevalence ranging from 67 to 100% [4]. Its time of onset can be variable in relation to myopathy, as was shown in a study conducted on a large Spanish cohort where 80 out of 145 anti-Jo1-positive patients (55.2%) presented with ILD at the time of the diagnosis, out of whom 33 (22.8%) had also associated myositis, whereas 47 (32.4%) had only lung involvement [5]. In addition, anecdotal cases and small case series of acute respiratory failure as initial manifestation were reported in the literature [6-9]. Therefore, even though in other cohorts the prevalence of amyopathic ASS was reported to be low [10,11], the still high percentage of patients without myositis symptoms at onset underscores the importance of considering ASS in the differential diagnosis of patients presenting with idiopathic ILD and with interstitial pneumonia with autoimmune features [12,13].

The most common radiological pattern found on high resolution computed tomography (HRCT) in anti-Jo1 patients is nonspecific interstitial pneumonia (NSIP), followed by organizing pneumonia and usual interstitial pneumonia (UIP); the most frequent elementary lesions seen are ground-glass opacities, interlobular septal thickening, reticulation and consolidations, whereas honeycombing is quite rare [4,10, 14-16].

No controlled studies are available to guide therapy in ASS. Manifestations other than ILD generally have an excellent response to corticosteroids alone. Glucocorticoids are the first-line agent for patients with associated ILD. The tapering regimen is prolonged, with the total duration determined by the disease course. ILD, despite a favorable initial response to steroids, frequently recurs. There is no consensus on the most effective steroid-sparing immunosuppressive agent or regimen. Agents that have been used with variable success have included cyclophosphamide, azathioprine, mycophenolate mofetil, cyclosporine, tacrolimus, intravenous immunoglobulin, and rituximab [4,13,17].

## Case presentation

A 68-year-old male presented with progressive shortness of breath over 2-3 weeks. His symptoms started two days after receiving pneumococcal and zoster (RZV, Shingrix) vaccines. He reported feeling quite well until his respiratory symptoms started. Initially, he had shortness of breath (SOB) with walking a couple of blocks; by the time he presented to our institution, he was short of breath while talking. He reported a mild cough with mild sputum production. He also endorsed night sweats, however no fever. The patient denied muscle weakness, muscle pain, joint pain, oral ulcers, skin rash, dysphagia, or Raynaud symptoms. His family history was remarkable for rheumatoid arthritis in his grandmother and sister; his son has alopecia. He denied any significant medical history. He denied recent sick contacts. His surgical history was notable for tonsillectomy and hernial repair. He is a former smoker who quit 35 years ago with ten pack-year smoking history.

He was evaluated initially at a different medical center where he presented with acute hypoxemic respiratory failure due to unclear etiology. He did not respond to initial management and had worsening oxygen requirements. He was transferred to our institution for further workup including evaluation for a lung transplant. Upon presentation to our institution, he was severely hypoxic; he required up to 60 liters 100% FIO2 of high-flow oxygen. His other vitals showed tachypnea with respiratory rate 22-30, temperature max 99.2, sinus tachycardia with heart rate 120, and normal blood pressure. His lungs were clear to auscultation bilaterally, with no rhonchi. Skin exam showed hyperkeratotic skin changes at fingertips consistent with mechanic's hand. His initial comprehensive metabolic panel (CMP) and complete blood count (CBC) were unremarkable except for leukocytosis. He tested negative for SARS-CoV-2 COVID-19 twice and bronchoalveolar lavage (BAL) was negative for infections.

**Figure 1:**
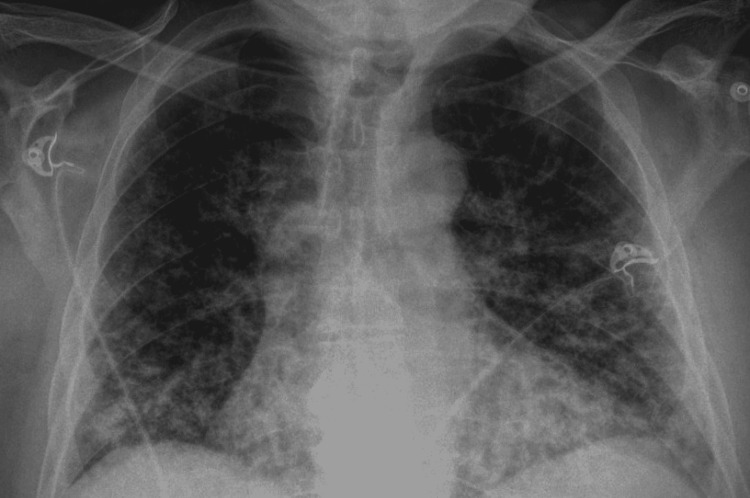
Initial Chest X-ray showing bilateral basilar lung infiltrates

**Figure 2:**
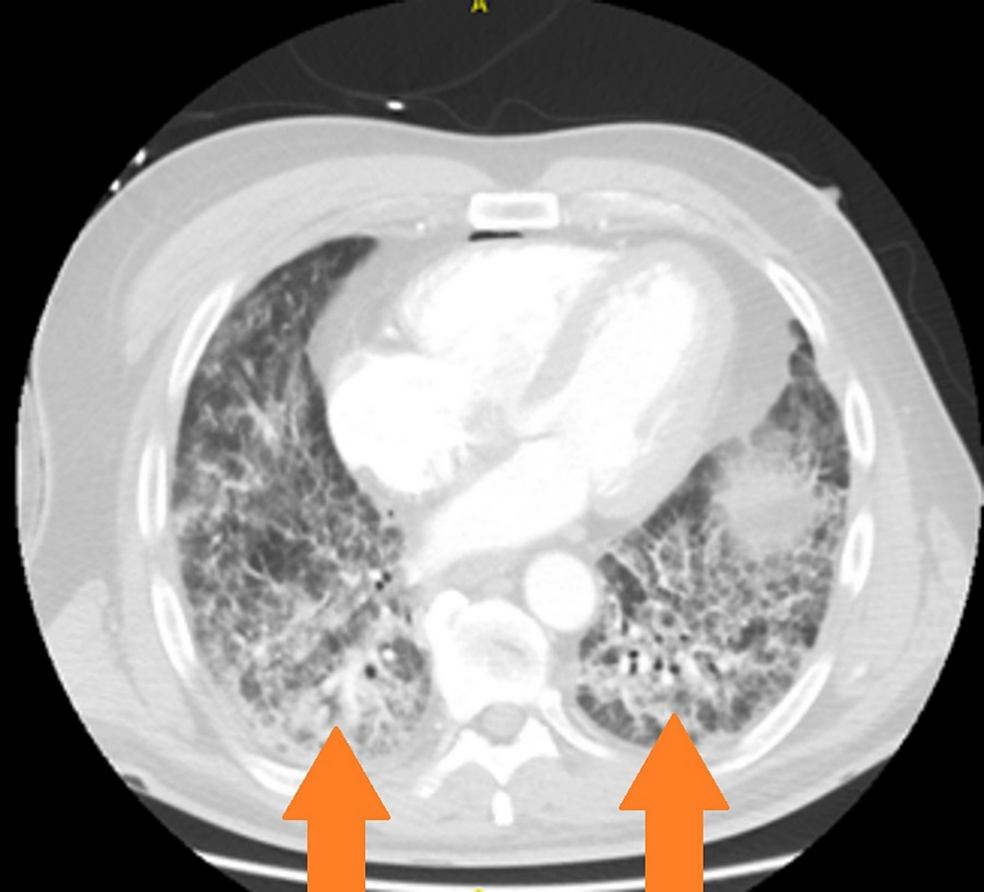
Initial chest CT scan showing dense bilateral basilar reticulations highlighted by the arrows

Chest X-ray (CXR) showed diffuse bilateral ground-glass alveolar and interstitial infiltrates with bibasilar prominence and mediastinal adenopathy, shown in Figure 1 below. Chest computed tomography (CT) scan showed findings consistent with pneumonitis, shown in Figure 2 below.

Further laboratory results were significant for elevated lactate dehydrogenase (LDH), alanine aminotransferase (ALT), aldolase and positive Anti-Jo-1 antibody, as shown in Table 1 below. His microbial cell-free DNA molecular testing (Karius) was negative.

**Table 1:** Serum laboratory test results including liver enzymes, auto-immune antibodies and tumor markers

Laboratory investigation	Reference range	Result
Alanine aminotransferase (ALT)	4-51 units/L	70
Aspartate aminotransferase (AST)	5-46 units/L	39
Alkaline phosphatase (ALP)	40-129 units/L	64
Lactate dehydrogenase (LDH)	60-200 units/L	465
Hepatitis-B core antibody (HBcAb)	-	Positive
Hepatitis B surface antigen (HBsAg)	-	Negative
Hepatitis C antibody	-	Negative
Creatinine Kinase (CK)	24-200 units/L	60
Aldolase	1.5-4.1 units/L	19.0
Anti-Jo-1 antibody	<1.0 units/L	1.1
Antineutrophil cytoplasmic autoantibodies (ANCA)	-	Negative
Anti-citrullinated peptide (CCP) antibodies	-	Negative
Carcinoembryonic Antigen (CEA)	0-5 units/L	6.9
Carbohydrate antigen 19-9 (CA-19-9)	0-35 units/L	37

His presentation raised suspicion for acute interstitial pneumonitis, most likely secondary to inflammatory myopathy. He received pulse dose methylprednisolone 1 g IV daily for three days, which was subsequently transitioned to prednisone 60mg daily, in addition to high dose Mycophenolate mofetil. He was also started on Atovaquone for Pneumocystis jirovecii (PJP) prophylaxis. He was receiving therapeutic doses of enoxaparin for a recent diagnosis of unprovoked deep vein thrombosis (DVT). He was switched to bivalirudin as a result of the finding of positive heparin antibodies. His CT chest showed low burden subsegmental pulmonary embolism, for which he underwent inferior vena cava (IVC) filter placement. Carcinoembryonic antigen (CEA) and Carbohydrate antigen 19-9 (CA-19-9) levels were elevated; however extensive evaluation including positron emission tomography (PET) scan and magnetic resonance imaging (MRI) abdomen did not reveal an underlying malignancy.

A diagnosis of acute interstitial pneumonitis most likely secondary to anti-synthetase syndrome was made based on positive anti-Jo-1 antibody, elevated aldolase, ALT, LDH, and mechanic’s hand features.

**Figure 3:**
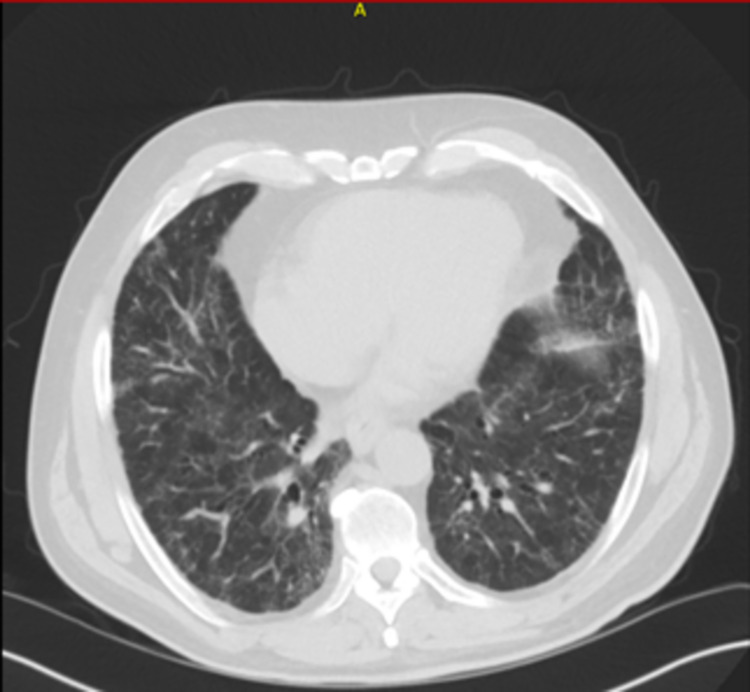
Follow-up chest CT scan six months after initiating treatment

The patient significantly improved with therapy; his oxygen requirements markedly decreased. He was eventually discharged from the hospital on minimal oxygen support. He participated in an outpatient pulmonary rehabilitation program during which he showed consistent improvement in physical endurance and was eventually completely weaned off oxygen. He remained on maintenance immunosuppressive therapy. His labs remained stable. His follow-up chest CT six months later demonstrated near-complete resolution of initial findings, as seen in Figure 3 below.

## Discussion

There has been only one published case report about the development of ASS after vaccination, and it described a patient who developed ASS one week after the administration of the influenza vaccine [18]. Nonetheless, vaccines including hepatitis B vaccine, bacillus Calmette-Guérin, tetanus, influenza, smallpox, poliomyelitis, diphtheria, diphtheria-pertussis-tetanus (DPT), and combination of diphtheria with ‘scarlet fever’ and of DPT with polio have been associated with polymyositis (PM) and dermatomyositis (DM) [19-26]. Yet, no case reports have previously described the development of idiopathic inflammatory myopathies after recombinant zoster vaccine (Shingrix) or pneumococcal polysaccharide vaccine (Pneumovax). In our case report, we describe for the first time an alleged temporal relationship between the administration of Pneumovax and Shingrix vaccines and the development of ASS.

Current literature reviews have failed to identify any strong evidence linking the administration of vaccines and the development of immune-inflammatory myopathies. Most if not all the existing evidence is revolving around case reports. More specifically, there is no statistically significant evidence in the literature for an increase in the incidence of DM or PM after any massive vaccination program, whether prospective or retrospective and no meta-analyses have been published to date [27]. Epidemiological studies and controlled studies to evaluate the possible role of vaccines in a variety of autoimmune diseases obtained negative results [28,29]. However, these types of studies lacked the statistical power to rule out an "extremely rare" causal relationship as also suggested by published case reports [28,30]. It seems not unreasonable to propose that vaccines rarely induce autoimmune reactions in presumed genetically or immunologically predisposed individuals [31], but there exists no “unequivocal and irrevocable” evidence of a causative link between vaccination and autoimmunity [32,33]. Even if some vaccines were shown to be associated with slightly increased incidence of some autoimmune diseases, it would not alter recommendations to adhere to vaccination programs to ensure the public’s health [27].

The development of vaccines was a significant contribution to public health in the modern era [34]. Vaccination is a powerful immune system stimulant that has the theoretical potential to induce or exacerbate immune disturbances that manifest as serological indices of immune system dysregulation or as clinically manifest autoimmune disease [35,36]. As infections do not overtly cause autoimmune disease in most individuals, it is the interplay of several factors which account for the development of autoimmunity. Hence, patients with a genetic predisposition for autoimmunity probably have an increased risk for post-vaccination autoimmune disorder. Theoretically, the more a vaccine is complex, and the array of its antigens varied, the more likely would it trigger an autoimmune response that may eventually turn into a full-blown autoimmune disease [37]. Yet, in the case of vaccines, these mechanisms are enhanced due to the presence of adjuvants, which are potent stimulators of the immune system [21,35].

## Conclusions

Due to the relative rarity of autoimmune conditions compared to vaccine administrations, and due to the wide variability of patients' ages, along with the varying durations of the reported latency periods, it's very hard to draw conclusions about whether vaccines play a role in the development of autoimmune conditions. However, it will remain an exciting area to conduct future studies to examine the potential role they can play in inducing autoimmune responses in genetically predisposed individuals.
